# Projected economic losses due to vector and vector-borne parasitic diseases in livestock of India and its significance in implementing the concept of integrated practices for vector management

**DOI:** 10.14202/vetworld.2018.151-160

**Published:** 2018-02-09

**Authors:** B. W. Narladkar

**Affiliations:** Department of Veterinary Parasitology, College of Veterinary and Animal Sciences, MAFSU, Parbhani - 431 402, Maharashtra, India

**Keywords:** economic injury levels, economic threshold levels, integrated pest management, vectors

## Abstract

Broadly, species of arthropods infesting livestock are grouped into flies (biting and non-biting), fleas, lice (biting and sucking), ticks (soft and hard), and mites (burrowing, non-burrowing, and follicular). Among which, biting and non-biting flies and ticks are the potent vectors for many bacterial, viral, rickettsial, and protozoan diseases. Vectors of livestock are having economic significance on three points (1) direct losses from their bite and annoyance, worries, and psychological disturbances produced during the act of biting and feeding, (2) diseases they transmit, and (3) expenditure incurred for their control. Flies such as *Culicoides* spp. and *Musca* spp. and various species of hard ticks play important role in disease transmission in addition to their direct effects. For control of vectors, recent concept of integrated pest management (IPM) provides the best solution and also addresses the problems related to acaricide resistance and environmental protection from hazardous chemicals. However, to successfully implement the concept of IPM, for each vector species, estimation of two monitory benchmarks, i.e., economic injury level (EIL) and economic threshold level (ETL) is essential prerequisite. For many vector species and under several circumstances, estimation of EIL and ETL appears to be difficult. Under such scenario, although may not be exact, an approximate estimate can be accrued by taking into account several criteria such as percent prevalence of vectors in a geographical area, percent losses produced, total livestock population, and current prices of livestock products such as milk, meat, and wool. Method for approximate estimation is first time described and elaborated in the present review article.

## Introduction

One of the greatest challenges of the 21^st^ century is the need to feed a growing population while improving the productive capacity of agricultural ecosystems and the health and integrity of surrounding environments for future generations [1]. For enhancing the crop production, indiscriminate use of hazardous chemicals and fertilizers leads to irreparable damage to the health of human, livestock, soil, and environment. In the recent decades, scientists across the globe alarmed about the consequences of this issue. Thus, to address this, enormous research has been done throughout the world, which emerged into a concept of integrated pest management (IPM) and organic production methods, which can work together to address this vital challenge. The integration of livestock into cropping, through draught power and manure, increases the area cultivated, improves the timeliness of agricultural operations, and helps to maintain soil structure and fertility [2], for this purpose, livestock rearing becomes the complementary and supplementary business to ­agriculture. Therefore, it is equally true that IPM practices are also necessary to achieve control of ectoparasites involving pests-cum-vectors for livestock.

To achieve the goal of optimum or maximum production from livestock, it is mandatory to increase the number of healthy livestock by scrupulously following good managemental practices, provision of balanced diet, and protection from infectious diseases. In addition to bacterial and viral diseases, protection from vectors and vector-borne diseases is an obligatory step and for which implementation of effective IPM practices is need of the hour. More recently, researches in the areas of various aspects such as physical and biological aspects of control of vector pests had taken a great momentum and are in full swing. By integrating all such aspects of control, there is strong need to develop an IPM module, for which, estimation of two monetary benchmarks, i.e. economic injury level (EIL) and economic threshold level (ETL) is not only prerequisite but also essential step [3]. These two benchmarks suggest the balance between losses accrued on the account of vectors and vector-borne diseases and expenditure incurred on vector control. However, many a time, assessment of exact estimation of EIL and ETL is not possible in a particular geographic area, if the exact estimation of EIL and ETL is not possible, the method proposed in the present review article for approximate calculation of economic losses can play a role of pathfinder and thus gains importance.

The present review article is highlighting the importance of IPM for vector-cum-pests of livestock and an alternative approach for approximate estimation of economic losses, by taking into account, epidemiological data in a particular geographic location and losses due to vectors.

## Vectors Infecting to Livestock

Broadly, species of arthropods infesting livestock are grouped into flies (biting and non-biting), fleas, lice (biting and sucking), ticks (soft and hard), and mites (burrowing, non-burrowing, and follicular). Among five major groups of ectoparasites, biting and non-biting flies and ticks are the potent vectors for many bacterial, viral, rickettsial, and protozoan diseases.

## What is the Concept of IPM?

Consequent to effects of global warming, resistance accrued to insecticides on their indiscriminate use, wrong animal husbandry practices and poor status of nutrition, and one or the other reasons, in the past few decades, prevalence of vectors-cum-pests and vector-borne diseases is on high rising trend. The World Health Organization declared on April 7^th^, 2014 on the occasion of world health day, the theme of the year as “Protect yourself from ­vector-borne diseases.” In conclusion, it is need of the hour to combat the vectors and protect our animals from vectors. The best way for combating the vectors is the implementation of IPM or integrated vector management practices suitable to local conditions.

Baker *et al*. [1] in his book narrated that both organic and IPM tactics require greater management skill to implement effectively than the calendar-based application of inputs. They also cited the definition of IPM given by USDA [4], which gives the impression about each aspect of IPM. According to USDA, IPM is “a science-based, decision-making process that identifies and reduces risks from pests and pest management related strategies. The IPM coordinates the use of pest biology, environmental information, and available technology to prevent unacceptable levels of pest damage by the most economical means while minimizing risk to people, property, resources, and the ­environment. IPM provides an effective strategy for managing pests in all arenas from developed agricultural, residential, and public lands to natural and wilderness areas. The IPM provides an effective, all encompassing, low-risk approach to protect resources and people from pests.” Thus, application of IPM is need of hour to avoid the health and environment related risks strongly posed by present strategies for pest control.

## Why IPM is Must?

### Advantages of IPM

The key benefits of IPM as highlighted by Baker *et al*. [1].

- “Reduces reliance on single tactics; improves the resilience of production systems.” It is an integration of methods such as physical, cultural, herbal, and biological without any reliance on hazardous chemical pesticides. Chemical pesticides are used as last resort when the population of pest will cross ETL and EIL levels.
- “Can reduce pesticide use, residues, pest damage, production costs and risks, and health and environmental impacts.” It helps to reduce the use of pesticides and thus protects the beneficial insect population along with health of human, livestock, soil, and environment.
- IPM is a “big tent” of fundamental principles with the strong advantage of flexibility to create new approaches for addressing any pest complex at different levels and thus it adapts to any production goals including the organic production.

### High consumption of pesticides in agriculture

According to the data presented by Pretty and Bharucha [5], global pesticide use has grown over the past 20 years to 3.5 billion kg/year. Further, they have highlighted the major quantum used in countries of the world. Accordingly, China, USA, and Argentina now account for 70% of world pesticide use in agriculture (2.44 billion kg/annum), with China alone now using half of the pesticides worldwide. Six countries each consume between 50 and 100 M kg (Thailand, Brazil, Italy, France, Canada, and Japan) and thirteen between 10 and 50 M kg (India, Spain, Germany, Bangladesh, Turkey, South Africa, Russia, Chile, Vietnam, UK, Ghana, Cameroon, and Pakistan). From the data, it can be concluded that India, along with other twelve countries, is ranking third in the consumption.

### Economics

Related to cost of pesticides and pesticide resistance: The external costs of pesticides are $4-$19 (€3-15) per kg of active ingredient applied [5], indicating the high cost of chemical pesticides, not economical to the farmers in the countries like India.

### Demand for organic food

Rising trend of demand for organic food: Demand for quality-assured products which are chemical free or organic food products.

IPM offers environmental friendly control against pests/vector, provided it is adapted in totality and not only by relying upon the single measure of using hazardous chemical pesticides.

## Economic Importance of Vectors and Vector-Borne Diseases of Livestock in Sustainable Agriculture

Vectors of livestock are having economic significance on three points: (1) Direct losses from their bite and annoyance, (2) diseases they transmit, and (3) expenditure incurred for their control. Flies such as *Culicoides* spp. and *Musca* spp. also play important role in disease transmission in addition to their direct effects.

*Tabanus* fly-transmitted disease trypanosomosis in buffaloes has a significant impact on the economics of the dairy farmers. Trypanosomosis directly constrains the productivity of cattle by reducing birth rates, increasing abortion rates, and increasing mortality rates [6]. Globally, ticks and tick-borne diseases (TTBDs) are the major constraints on profitable livestock production and productivity. Hemoprotozoan diseases, especially babesiosis, anaplasmosis, theileriosis, and trypanosomosis are considered as the major impediments to the health and productive performance of cattle [7]. Apart from the direct effects, the most important feature of ticks is that they are vectors, as well as reservoirs, of multiple pathogens. Ticks and tick-transmitted parasites have co-evolved with various wild animal hosts, being part of the ecosystem’s equilibrium [8]. TBDs, long known but often neglected, are progressively being recognized not only due to their economic impact on livestock but also due to their impact to human health, to which they have become a threat.

Tick-borne diseases cause substantial losses to the livestock industry throughout the world [9] as these have got a serious economic impact due to obvious reason of death, decreased productivity, lowered working efficiency [10], and increased cost for control measures [11]. The TTBDs have been recognized as a major cause of production loss predominantly in tropical and subtropical countries of the world [12-16]. According to the FAO [17], 80% of the world’s cattle population is exposed to tick infestation and has estimated the impact of 7.3 US $/head/year. Loss of ­appetite in heavily tick-infested cattle was found responsible for 65% of the body weight reduction [18]. The cattle tick *Rhipicephalus microplus* causes economic losses to the Brazilian cattle industry estimated at U$3.24 billion per year [19]. Table-1 [20-33] explains how pests of livestock are having economic importance.

**Table-1:** Effect of vectors-cum-pests on production from livestock resulting in a reduction in economic gains.

Pest species	Economic losses
House flies	3.3% decrease in milk production [20]
Stable flies	5% decrease in milk production [21]
Tabanid flies	0.08-0.10 Kg loss of weight per day when animal exposed to 66-90 flies. Decreased feed efficiency by 16.9% [22]
*Culicoides* spp. (Biting midges)	Transmission of bovine ephemeral fever (loss of draught work), BT outbreaks results in 6 million US dollar loss [23]
*Simulium* spp.(Black flies)	Severe morbidity and mortality due to attack by swarms [24], Reduced milk production and weight gain by 50% [25]
Mosquitoes	Annual loss in terms of production and control costs amounting to 5 million US dollar annually [26] in the USA
*Haematobia irritans* (Horn fly)	Decrease weight gain and milk production [27]. Increased weight in fly free animals by 1.06 kg/day [28]. In Australia, third most costly disease to cattle producers with the average loss of production around $30 per head per year. Total national economic impact is estimated to be over $98.7 million a year [29]
Lice	In HF calves, weight losses up to 9.1 kg [30]. Total annual losses in American cattle from lice US $126.3 million [21]
Mange mites	Decreased feed conversion efficiency, reduced milk production of 10-15%
Cattle grubs	Reduced milk flow and reduced weight gain. Excessive trimming of hides
Ticks	$ 275.7 million annual loss [21] Transmission of tickborne diseases and cost of tick control. The index in which each engorged female would be responsible for 8.90 mL of milk reduction [31]. Estimated reductions of up to 50% of milk production on dairy farms were related in the available literature [32]
*B. microplus*	Australia US$ 62 million [33]

## Importance of Estimation of Projected Economic Losses

It is essential for:

- It is a best approximate estimate of EIL. In implementing IPM practices against the particular pest, a basic monetary benchmark to be taken into account is EIL. The data obtained after calculation of such projected economic losses will be highly useful for work out the losses caused by a single pest and its cost of control.
- It will help in decision-making about real-time need for control of a pest in a particular geographic area. If losses are less than the cost of control, accordingly decision about control measures to be adapted can be taken.
- Data generated from such estimations will be of great value to appraise the policy decision makers such as government agencies, NGOs, and private pharmaceutical companies about the research and control programs.
- In the preamble of many research proposals and in the introductory part of many research articles, to quote the importance of particular parasitic species, it is customary to write huge losses or it is also mentioned that certain million or billion US $ losses per annum. The exact meaning of such huge losses cannot be assessed. Similarly, it is also beyond understanding about how the cited value of million or billion US $ has been assessed. Thus, estimation of projected economic losses will be helpful in citing at least approximate losses occurring on the account of infection of particular parasitic species in question.
- To inform the farmers/milk producers about ­probable losses, such kind of data are highly useful during extension activities.
- Similar data can be generated for helminth parasites of livestock for judging the EIL levels for effective implementation of integrated parasite control.

## Method of Estimation of Projected Economic Losses

It shall be first to understand that all these estimates are approximate/projected and exact losses can never be possible to work out. Similarly, it is also to be taken into account that certain yardstick is essential for measuring the probable losses, which can be achieved through estimating the projected economic losses. The factors to be taken into account for estimation are (a) data on total population of a particular geographic area of a particular species of animal, age, sex, milking or non-milking, etc., (b) percent population of a particular animal species affected with the particular pest in a geographic area. Such data can be gathered by retrieving literature about percent prevalence studies undertaken by various research workers, (c) current market rate (CMR) of animal products such as meat/milk/wool, and (d) total sum of losses from particular pest(direct) or from disease transmission or from milk/meat/wool. It can be obtained either from scientific literature available on such parameters or it can be simply worked out by taking into account probable loss and cost of treatment or control. The approximate estimate of losses can be estimated by simple algebraic calculations with following mathematical equation.


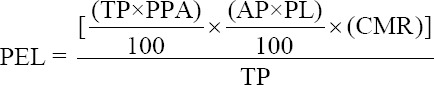


Where in,

PEL: Projected economic losses from a particular vector/vector-borne disease in a geographical area per animal or from total livestock population.

TP: Total population exposed to vector/at risk in a geographical area.

PPA: Percent population affected/exposed/at risk of vector/vector-borne disease in a geographical area.

AP: Standard average production of milk/meat/wool from individual/from total livestock population animal in a geographical area.

PL: Percentage loss from vector/vector-borne disease an individual animal in terms of milk/meat/wool.

CMR: Current market rate per unit of milk/meat/wool.

By taking example cited in Table-2 [20,21,23,26,34-46], above formula can be well explained.

**Table-2:** Projected economic losses from important vectors-cum-pests of livestock in India.

S.No.	Species of the pest	Direct losses estimated	Total milk^#^ production in India	Projected loss of milk production	Projected loss in ₹	Any other losses estimated
1	House flies (non-biting fly)	3.3% decreased milk production [20]	Total 132.43 million tons during 2012-13 in India	@3.3%-4.37 million tones	@₹ 38 L/Kg = ₹ 16606 crore per annum	Public health and public nuisance [34]. Unsanitary milk production [26]
2	Biting midges Culicoides (biting and bloodsucking fly)	Transmission of bovine ephemeral fever (loss of draught work), bluetongue outbreaks result in 6 million US dollar loss [23] Total 18.97% loss of milk production [36]	Total 132.43 million tons during 2012-13 in India	@18.97%-25.12 million tones	@₹ 38 L/Kg = ₹ 95463 crore per annum	BT disease has special status which restricts free trade of animals accounts for 125 million US Dollar revenue annually [35]
			7.02 L/day crossbred cow	@18.97%1.33 L/day loss	₹ 50.54 cow/day	
			2.36 L/day indigenous/ND cow	@18.97%0.45 L/day loss	₹17.10 cow/day	
			4.80 L/day Indian buffalo	@18.97%0.91 L/day	₹35.48 buffalo/day	
3	Ticks (many species)	$ 275.7 million annual loss due to transmission of tick-borne diseases and cost of tick control [21] Reduction of 23% in milk yield/day crossbred Holstein-Zebu cows [37] Reduction of 529 kg (26%) of milk/lactation in Holstein cows [38] Total losses and control of babesiosis and anaplasmosis in India costs, 57.2 million US dollars annually In India, almost all the livestock species suffer from tick infestations India alone the cost of TTBDs in animals has been estimated to the tune of US$ 498.7 million (more than 2000 crore) per annum [39]	Total 132.43 million tons during 2012-13 in India	@23.0%-30.46 million tones	@₹ 38 L/Kg = 115748 crore INR ₹ per annum	Tick pyemia, tick toxicosis, tick paralysis TTBD’s have been implicated to cause projected loss of about $500 million annually [40-43] Production losses in dairy cows due to tick infestation are estimated to be 8.9 mL of milk and 1 gram live weight gain per engorging female tick per day [44] In Australia,[45] estimated that an animal with an average of 40 ticks/day could lose weight equivalent to 20 kg/year
			7.02 L/day CB cow	@23%-1.61 L/day loss	₹ 61.18 cow/day	
			2.36 L/day/indigenous/ND cow	@23%-0.54 L/day loss	₹ 20.52 cow/day	
			4.80 L/day/ Indian buffalo	@23%-1.10 L/day loss	₹ 41.80 buffalo/day	

# Total milk production and average milk production per individual animal are referred from Annual Report 2013-14, Government of India [46]. For calculation of total loss in terms of INR (₹), rate of milk/L is considered as average ₹38/L for buffalo/cow milk

Example 1: (For calculation of projected economic losses for total livestock population).

Total livestock population of total milking cows + buffaloes in India is (TP): 80526070 (8.05 crore) [47].

Percent population affected (PPA) is: 100%.

Standard Average/total milk production of India from total milking cows+buffaloes (AP): 132.43 million tons (13243 core liters) [46].

Percent loss from vector/vector-borne disease to animal in terms of milk (PL):18.97% [36].

Current market rate per unit of milk (CMR): ₹ 38.

If these values entered in the above formula, the calculations are…


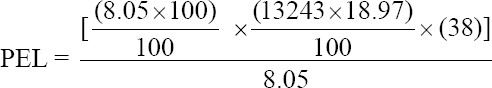


PEL projected economic loss from total cattle and buffalo milking animals population in India per year = ₹95463.48 crore.

Example 2: (For calculation of projected economic losses for individual animal).

Total livestock population of India is (TP): 1.

Percent population affected (PPA) is: 100%.

Standard average total milk production of India from TP is (AP): 7.02 L per crossbred cow [46].

Percent loss from vector/vector-borne disease to animal in terms of milk (PL):18.97% [36].

Current market rate per unit of milk (CMR): ₹ 38.

If these values entered in the above formula, the calculations are…


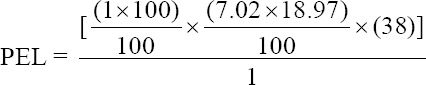


PEL per individual animal = ₹ 50.60 per crossbred cow per day.

Table-3 [12,39,41,47-52] narrates estimated losses in the literature, while Tables-4 and 5 [46,47,52-61] exemplify the estimation of approximate projected economic losses by citing the examples of five states.

**Table-3:** Losses due to TTBD reviewed from the literature.

S.No.	Disease transmitted by vector	Losses	Reference	Losses as on today in October 2017 $ [51]	Losses in ₹ in October 2017* (1US$=₹ 65.07 on 18.10.2017)
1	In India tropical theileriosis	Annual loss US$ 800 million	[48]	US$1295 million	₹ 8426.7 crore
2	A recent estimate calculated the costs of control of TTBDs affecting Indian livestock as	498.7 million US $ per annum	[41]	US$668.96 million	₹4353.0 crore
3	Total losses due to surra per animal in ND cow, CB cow, and buffalo	ND:₹ 3, 328.18, CB:₹ 6, 193 and buffalo: ₹9,872.33	[49]	ND: ₹183.86 crore CB: ₹ 713.5 crore buffaloes: ₹ 311.21 crore	ND:₹ 183.86 crore CB: ₹ 713.5 crore buffaloes: ₹ 311.21 crore
4	India suffers losses due to babesiosis in livestock	57.2 million US dollars annually	[50]	US$ 84.74	₹ 551.54 crore
5	In India alone, TTBD’s have been implicated to cause projected loss	$500 million annually	[39]	US$ 595.20	₹ 3873.06 crore
6	As per the 1997 estimates, the global production loss caused by TTBDs	13.9–-18.7 billion US $ annually	[12]	21.38–28.76 billion US $ annually	

* https://www.exchange-rates.org/Rate/USD/INR

**Table-4:** Projected economic losses due to trypanosomosis in buffaloes in India.

S.No.	State	Reference	% Prevalence	Total ^#^population [47]	Affected population	Loss of milk per affected animal in ₹ [49]	Total loss in ₹	Loss per animal in ₹
					
			a	b	a×b/100=c	d	c×d = e	e/b
1	Andhra Pradesh	[52]	7.28	9272257	675020	9872.33	666.40 crore	718.70
2	Chhattisgarh	[53]	22.03	600463	132281	9872.33	130.59 crore	2174.8
3	Karnataka	[54]	12.9	3110131	401206	9872.33	396.08 crore	1273.5
4	Punjab	[55]	9.35	4626033	432534	9872.33	427.01 crore	923.06
5	Overall India	[56]	2.69	92599075	2490915	9872.33	2459.1 crore	286.2

# 19th Livestock Census [47]

**Table-5:** Projected economic losses due to theileriosis in CB cattle in five states of India

S.No.	State	Reference	% Prevalence	Total ^#^population	Affected population	Loss of milk [41]	Rate	Total loss of milk	Total loss in ₹ in crore	Loss per animal in ₹
							
			a	b	a×b/100=c	d	e	c×d = f	e×f = g	g/b
1	Gujarat	[57]	37.0	1734161	641639	127	38	81488143	309.65	1785.62
2	Karnataka	[58]	17.7	2707335	479198	127	38	60858182	231.26	854.20
3	Kerala	[59]	16.0	1115375	178460	127	38	22664420	86.12	772.16
4	Tamil Nadu	[60]	13.0	5467646	710794	127	38	90270838	343.02	627.38
5	Uttarakhand	[61]	45.4	416977	189307	127	38	24042058	91.35	2191.00

d=Loss of milk per affected animal in liter, e=Rate of milk per liter considered as ₹ 38,

# 19th Livestock Census [47]

## Recent Developments in IPM

After 1970, IPM concept in agriculture against crop pests has emerged and flourished to the best possible extent; however, it is at infancy stage against pests of livestock, the reasons could be (a) difficulty in estimating ETL and EIL levels of pest infestation to livestock, (b) very few studies available on mapping of pests in India, (c) poor network of extension activities failed to understand livestock owners about implementation of IPM, and (d) differences in application of IPM tactics from crop pests to livestock pests. Crop pests can be directly attacked on standing crop, while livestock pests have to be attacked simultaneously at their breeding places (off-host) and on animal body (on-host). As a result, IPM against livestock pests appears as difficult and needs rigorous efforts. However, it has become mandatory to adapt practices of organic farming and IPM in the sustainable agriculture.

Recent trends used for IPM of livestock pests include biological (use of pathogen and predators such as bacterial and fungal agents and herbal pesticides), cultural (provision of good health to animals for resisting the pests), mechanical (grooming combs, flea combs, and electrical devices), physical (sticky fly paper and fly proof net shed), sanitation and habitat modification (cleanliness of stables and animal houses and caulking of cracks and crevices), legal quarantine measures (quarantine of sick and pest infected animals for avoiding the spread of pests), and last resort as use of chemical pesticides. Recently, push–pull mechanism based on semiochemistry has been evolved as emerging trend. In which the use of semiochemicals for control of cattle brown ear tick, use of kairomone trap for control of tsetse flies, and zooprophylaxis using animals for control of malaria transmitted by *Anopheles arabiensis* mosquitoes are the some of the examples.

To update recent developments in the areas of IPM, IPM practices are exemplified for two representative major vectors of livestock, one representing flies group (Diptera) and another representing Acarina group.

### Culicoides midges

Physical: Provision of Net shed [62,63].

Modeling of midge population and prediction models: More extensive modeling of *Culicoides* biting midge populations in different geographical contexts will help to optimize control strategies and predictions of disease outbreaks [64-67].

### Biological

- *Metarhizium anisopliae* [68,69], *Metarhizium* is not toxic to mammals and environmentally friendly biological control agent for *Culicoides brevitarsis* [70].
- *Bacillus cereus* (CWBI-B1082) [36,71].
- *Bacillus thuringiensis* var *Israelensis*, *Bacillus sphaericus, Bacillus weihenstephanensis* WSBC, and *B. weihenstephanensis* KBAB4 [72].
- Predacious guppy fishes: *Poecilia reticulata* [73].
- Herbal control with Neem and Karanj oil [74].
- Animal shelter management [75].
- Mechanical control through habitat modification [76].
- *Culicoides* species becoming less susceptible to deltamethrin thus need of alternative control [77].
- Integrated management of *Culicoides* spp. [78].

### Rhipicephalus (B.) microplus

#### Physical

Caulking process involving burning of tick eggs in the cattle shed for the annihilation of breeding places to be undertaken 3 consecutive times at weekly interval in a season [79], by slow burning over 1 or 2 days [80].

#### Biological

- *Verticillium lecanii* and *Beauveria bassiana* strain LBBb-14 (applied on calves body) [81].
- *In vitro* efficacy of *B. bassiana* in engorged females [82].
- Strain Ma-z4 of *M. anisopliae* and strain Bb-1 of *B. bassiana* (applied directly to animal body) [83].
- Use of three herbal oils: Neem oil (*Azadirachta indica*), karanj oil *(Pongamia* *pinnata*), and Nilgiri oil (*Eucalyptus globulus*) [36]. Alcoholic extracts of sitaphal (*Annona squamosa*) and neem (*A. indica*) against different life stages [39]. Crude extracts of *Allium sativum* cloves and *Carica papaya* seeds [84], use of herbal preparations used in ethnoveterinary and as green-fabricated nanoparticles as novel approach [85].
- Fungal control: *M. anisopliae* and *B. bassiana* [86-90]; *M. anisopliae* and *B. bassiana* gain high value in biological control of ticks because both the funguses exhibited the strongest anti-tick pathogenicity [91-93].
- Pheromones used by ticks for aggregation and mating can be artificially used in combination with acaricides [94].
- Use of oil formulation of funguses: The use of oil formulations containing these entomopathogens may increase the conidia stability and extend their persistence in the field protecting fungi against heat stress, desiccation, and particularly ultraviolet irradiation [95-99].

#### Tick vaccine

Subolesin as a candidate vaccine antigen for the control of cattle tick infestations in Indian situation [100].

## Conclusion

Vectors and vector-transmitted diseases in livestock pose high economic losses and need to be addressed using recent tool in the form of well-known concept of IPM. To successfully implement the concept of IPM, for each vector species, estimation of two monitory benchmarks, i.e. EIL and ETL is essential prerequisite. Accurate estimation of these two benchmarks appears to be difficult for almost all vector species infecting and transmitting the diseases in livestock. Under such scenario, a method for approximate estimation, first time, described and elaborated in the present review article has immense utility. The method described is based on the consideration of all important key factors such as percent prevalence of vectors in a geographical area, percent losses produced, total livestock population and current prices of livestock products such as milk, meat, and wool, and thus, economic losses estimated are close to the accuracy. Therefore, the method described is recommended for field utility.

## Authors’ Contribution

BWN is the sole author.
